# Mergeomics: multidimensional data integration to identify pathogenic perturbations to biological systems

**DOI:** 10.1186/s12864-016-3198-9

**Published:** 2016-11-04

**Authors:** Le Shu, Yuqi Zhao, Zeyneb Kurt, Sean Geoffrey Byars, Taru Tukiainen, Johannes Kettunen, Luz D. Orozco, Matteo Pellegrini, Aldons J. Lusis, Samuli Ripatti, Bin Zhang, Michael Inouye, Ville-Petteri Mäkinen, Xia Yang

**Affiliations:** 1 Department of Integrative Biology and Physiology, University of California, Los Angeles, Los Angeles, CA USA; 2 Center for Systems Genomics, University of Melbourne, Melbourne, Australia; 3 School of BioSciences, University of Melbourne, Melbourne, Australia; 4 Institute for Molecular Medicine, Helsinki, Finland; 5 Department of Molecular, Cell and Developmental Biology, University of California, Los Angeles, Los Angeles, CA USA; 6 Department of Medicine, David Geffen School of Medicine, University of California, Los Angeles, Los Angeles, CA USA; 7 Department of Genetics and Genomics Sciences, Icahn School of Medicine at Mount Sinai, New York, NY USA; 8 Department of Pathology, University of Melbourne, Melbourne, Australia; 9 South Australian Health and Medical Research Institute, Adelaide, Australia; 10 School of Biological Sciences, University of Adelaide, Adelaide, Australia; 11 Computational Medicine, Faculty of Medicine, University of Oulu and Biocenter Oulu, Oulu, Finland; 12 Insitute for Quantitative and Computational Biosciences, University of California, Los Angeles, Los Angeles, CA USA

**Keywords:** Mergeomics, Integrative genomics, Multidimensional data integration, Functional genomics, Gene networks, Key drivers, Cholesterol, Blood glucose

## Abstract

**Background:**

Complex diseases are characterized by multiple subtle perturbations to biological processes. New omics platforms can detect these perturbations, but translating the diverse molecular and statistical information into testable mechanistic hypotheses is challenging. Therefore, we set out to create a public tool that integrates these data across multiple datasets, platforms, study designs and species in order to detect the most promising targets for further mechanistic studies.

**Results:**

We developed Mergeomics, a computational pipeline consisting of independent modules that 1) leverage multi-omics association data to identify biological processes that are perturbed in disease, and 2) overlay the disease-associated processes onto molecular interaction networks to pinpoint hubs as potential key regulators. Unlike existing tools that are mostly dedicated to specific data type or settings, the Mergeomics pipeline accepts and integrates datasets across platforms, data types and species. We optimized and evaluated the performance of Mergeomics using simulation and multiple independent datasets, and benchmarked the results against alternative methods. We also demonstrate the versatility of Mergeomics in two case studies that include genome-wide, epigenome-wide and transcriptome-wide datasets from human and mouse studies of total cholesterol and fasting glucose. In both cases, the Mergeomics pipeline provided statistical and contextual evidence to prioritize further investigations in the wet lab. The software implementation of Mergeomics is freely available as a Bioconductor R package.

**Conclusion:**

Mergeomics is a flexible and robust computational pipeline for multidimensional data integration. It outperforms existing tools, and is easily applicable to datasets from different studies, species and omics data types for the study of complex traits.

**Electronic supplementary material:**

The online version of this article (doi:10.1186/s12864-016-3198-9) contains supplementary material, which is available to authorized users.

## Background

Most non-communicable diseases stem from a complex interplay between multiple genes, transcripts, proteins, metabolites and cumulative exposure to environmental risk factors [1]. In recent years, the advance of omics technologies has greatly enhanced our ability to measure the patterns of molecular entities and interactions at genome-scale. Public data repositories such as dbGaP for population-based genetic datasets [2] and Gene Expression Omnibus and ArrayExpress for gene expression and epigenomics datasets [3, 4] are continuously expanding with new experiments, and data acquisition projects such as ENCODE and GTEx are generating multidimensional datasets on the regulatory processes that link DNA variation with intermediate molecular traits and, ultimately, physiological or pathophysiological phenotypes [5–7]. Genome-wide association studies (GWAS), transcriptome-wise association studies (TWAS), epigenome-wide association studies (EWAS) and metabolome- and proteome-wide association studies have become commonplace in modern biomedical research. Therefore, data integration and interpretation has emerged as a new bottleneck on the road to discovery.

The combination of multiple omics studies is appealing, since a single genomic dataset is unlikely to provide deep mechanistic insight. Instead of one obvious candidate, most omic-wide studies produce a pattern of univariate statistical signals without a clear indication of what would be a suitable target for interventions [8, 9]. However, by integrating different types of data, converging patterns usually emerge and the search space for possible mechanisms can be greatly reduced. For instance, simultaneous measurement of DNA and RNA (genetics of gene expression) allows investigators to see if a particular genetic variant affects the downstream expression of a gene [10, 11], and functional data such as transcription factor binding, epigenetic modification or protein regulation from the ENCODE project [12, 13] can be used to further focus on the most promising candidates.

Multi-dimensional data integration has been previously addressed by pathway-based tools such as MAGENTA [14], iGSEA4GWAS [15], SSEA [16], and other network-based methods such as WGCNA [17], postgwas [18], dmGWAS and EW_dmGWAS [19], DAPPLE [20], NetWAS [21], and MetaOmics [22] have been developed to identify the biological processes (e.g. pathways) and specific genes or molecules that may be involved in pathogenesis. However, the available methods are typically tailored for a particular application area (e.g. human genetics with gene expression, protein-protein interactions or metabolomics) and may not be suitable for cross-comparison of results across diverse data types. In addition, the majority of the network tools start from a limited set of known top loci or genes, but it may be necessary to include the complete genome-wide patterns of signals for maximum sensitivity. Commercial tools such as Ingenuity (http://www.ingenuity.com) have been available for pathway and network analysis of different types of omics data such as gene expression and genetic data. However, these tools are not open source, hence limiting the accessibility by individual users and lacking the availability of detailed underlying algorithms and proprietary information. Additionally, the commercial tools usually do not provide the flexibility to incorporate different types of networks and pathways. For example, Ingenuity networks are primarily based on gene-gene relationships derived from literature rather than data-driven, tissue-specific network patterns the users may wish to use. For these reasons, we developed Mergeomics, an open source software to deliver flexible multi-omics integration, to identify pathways and model molecular networks of diseases, and to pinpoint promising targets for further experiments in a streamlined, generic and high-throughput manner.

In this report, we describe the main features of Mergeomics, and present simulations and case studies to demonstrate its performance. Mergeomics is the first publicly available implementation of a proven integrative methodology [23]. It employs two broad areas of analysis: Marker Set Enrichment Analysis (MSEA) identifies disease-associated biological processes via integration of omics-disease association and functional genomics data, and weighted Key Driver Analysis (wKDA) determines the key drivers that are suitable for targeted interventions to these processes. Here, we introduce new algorithms (hierarchical permutations and adaptive test statistics) and new concepts (co-hubs and weighted key drivers) to improve the applicability and performance over previous applications. We also report a case study on circulating cholesterol that shows how multiple human studies can be combined, and another case study on glucose regulation that demonstrates analysis across data types (genome, transcriptome and epigenome) and species (human and mouse). The source code for Mergeomics is available in Bioconductor (https://www.bioconductor.org/packages/devel/bioc/html/Mergeomics.html).

## Results and discussion

### Overview of Mergeomics

**Fig. 1:**
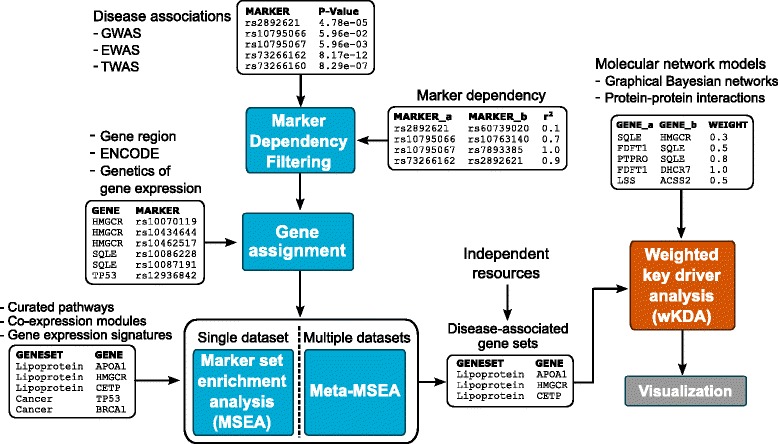
Main modules, data flow between them and examples of data types that can be integrated by Mergeomics

**Fig. 2:**
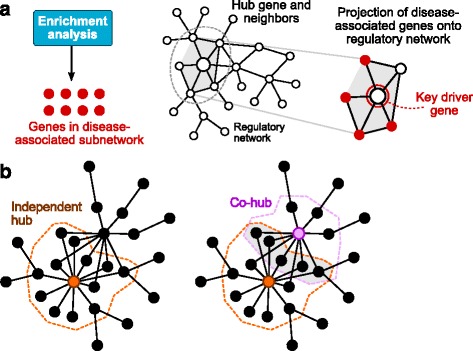
Schematic illustration of the concept of a key driver gene (**a**) and local hubs with overlapping neighborhoods (**b**)

Figure 1 shows the information flow within the Mergeomics pipeline. The Marker Set Enrichment Analysis (MSEA) combines disease association data (e.g., GWAS, EWAS, TWAS) of molecular markers (e.g., genetic, epigenetic and transcript variants), functional genomics data from projects such as GTEx and ENCODE, and pre-defined sets of connected genes. The output from MSEA is a ranked list of gene sets. We collectively denote these gene sets – which can be metabolic and signalling pathways, co-expression modules or gene signatures – as ‘disease-associated gene sets’. When multiple datasets of the same data type or different data types are available for a given disease or phenotype, the meta-MSEA component that is based on the same principles as MSEA but performs meta-analysis at the gene set level can be utilized. The Weighted Key Driver Analysis (wKDA, on the left in Fig. 1 and detailed in Fig. 2) was developed to identify local hubs in a gene network whose neighbours are enriched for genes in the disease-associated gene sets. Henceforth these hubs are referred to as key drivers.

### Marker set enrichment analysis

#### Rationale and design

MSEA is based on the idea that a collection of multiple associations is likely to contain true causal variants even if causality cannot be reliably established by univariate analysis. For instance, if multiple genes in a pathway are implicated, then the pathway as a whole is likely to be causal even if some of the gene signals were false positives. The primary inputs for MSEA include 1) marker to disease association statistics, where markers can be SNPs from GWAS, genes or transcripts from microarrays or RNA sequencing, epigenetic markers from DNA methylation profiling, metabolites from metabolomics or proteins from proteomics; 2) assignment of markers to their functional downstream target, and 3) sets of functional units of genes that co-operate or interact to perform a biological function or process.

MSEA starts with the conversion of a gene set representing a functional unit into a marker set. The corresponding disease association value for each marker is then collected for analysis. In most cases, the association *P*-values are used. If there are a large number of small *P*-values in the marker set compared to what can be expected by chance, we conclude that the gene set we have started from is enriched for disease associations (technical details in Methods). Each step in MSEA is fully customizable: it allows 1) association studies of different types or species; 2) different methods of marker-gene assignments, including expression quantitative trait loci (eQTL), transcription factor binding or sequence-proximity to regulatory or coding sequences; 3) filtering based on user-supplied confounding dependencies such as linkage disequilibrium between genetic markers; and it also utilizes 4) a non-parametric test statistic with multiple user-definable quantile thresholds to automatically adapt to a diverse range of association study datasets with different sample sizes and statistical power. For added applicability, MSEA runs a hierarchical gene-based permutation strategy to estimate null distributions that adjusts for shared markers between genes and gene size.

#### Parameter optimization

To test the performance and identify optimal parameters of MSEA, we performed simulation tests based on three cholesterol GWAS of varying sample sizes (a Finnish study of 8330 individuals [24], the Framingham Heart Study with 7572 participants [25], and GLGC with 100,184 people [26]) and a set of known causal lipid homeostasis genes from the Reactome pathway R-HSA-556833, “Metabolism of lipids and lipoproteins”. We resampled genes from this pathway into positive control signals of different magnitudes, and generated negative control signals from the gene pool excluding known cholesterol genes. This procedure was repeated 100 times, and performance was evaluated as sensitivity, specificity and positive likelihood ratio, as described in Methods.

We identified two important parameters, the percentage of top markers included and the threshold for confounding marker dependencies, that affect the performance of MSEA (Additional file 1: Table S1). The signal to noise ratio typically improved when genetic loci with relatively stronger associations (e.g., top 50 % markers) rather than the full association sets were used (Additional file 2: Figure S1). This confirms previous findings for complex traits that variants in the extremely weak association spectrum add noise and contribute little to disease biology [27]. For GWAS, linkage disequilibrium is a source of confounding marker dependencies. MSEA was less sensitive to LD thresholds for better powered studies such as the GLGC GWAS but smaller studies benefited from less stringent LD cutoffs, presumably due to improved statistical power (Additional file 2: Figure S1). Overall, we chose to use the top 50 % of GWAS loci, and an LD cutoff of r^2^ < 0.5 as the default setting for GWAS studies. Of note, differences due to datasets were typically larger than those due to parameters (Additional file 2: Figure S2) or variations in marker to gene assignment criteria (Additional file 1: Table S1).

#### Performance comparison with previous methods

**Fig. 3:**
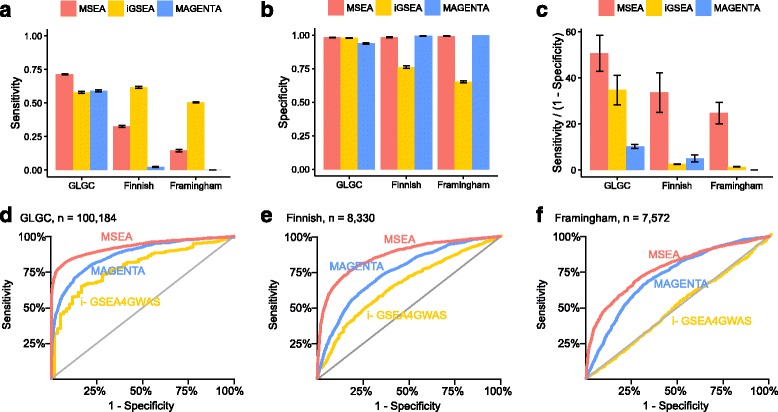
Comparison of three pathway enrichment methods across three GWAS. Performance is evaluated by sensitivity (**a**), specificity (**b**), positive likelihood ratio (sensitivity/(1-specificity)) (**c**) and receiver operating characteristic curve (**d**–**f**). Sensitivity was defined as the proportion of positive control pathways detected at FDR < 25 %. Specificity was defined as the proportion of negative controls rejected at FDR ≥ 25 %. Error bars denote the standard error of simulation results

We compared the performance of MSEA to MAGENTA [14] and i-GSEA4GWAS [15], two widely used implementations of gene enrichment analysis [28]. Compared to these methods, MSEA differs in test statistics, confounder adjustment and flexibility in data input. The same simulated positive and negative control pathways that were used for calibrating MSEA were also used to compare the three different methods (Fig. 3). The results are similar across all three total cholesterol GWAS: i-GSEA4GWAS lacked specificity and MAGENTA lacked sensitivity, whereas MSEA provided the best balance and receiver operator characteristics. The results remain robust when different false discovery rate (FDR) cutoffs were used (Additional file 2: Figure S3). Notably, the superior performance of MSEA over the other two established methods is more obvious when the GWAS involved smaller sample size (the Finnish and Framingham studies compared to GLGC) or heterogeneous population (the Framingham study compared to the more homogenous Finnish cohort), making MSEA useful for all types of studies including the underpowered ones. As the above performance comparison based on simulated positive and negative controls may give an unfair advantage to MSEA due to optimized calibration towards the positive controls, we performed additional tests with 1346 canonical pathways curated by Reactome [29], BioCarta (http://cgap.nci.nih.gov/Pathways/BioCarta_Pathways) and KEGG [30] (Additional file 1: Table S2). Consistent with results from the simulation approach, MSEA captured the largest number of true positive signals (calculated as the number of overlapped significant pathways among all three GWAS, minus the expected number of overlapped pathways from random gene sets).

### Meta-MSEA: gene-set level meta-analysis of multiple association studies

#### Rationale and design

For a disease phenotype, it is typical that multiple association studies of either the same data type (e.g., multiple GWAS) or different data types (e.g., an EWAS plus a TWAS) are available. Aggregating multiple studies of the same disease is an appealing strategy to increase signal-to-noise ratio, but marker-level integration is usually complicated by technical challenges. Therefore, we developed Meta-MSEA, which performs gene set-level meta-analysis of multiple association studies to avoid the need to match data platforms, species or ethnicity, an advantage not present in previous methods.

#### Performance evaluation

**Fig. 4:**
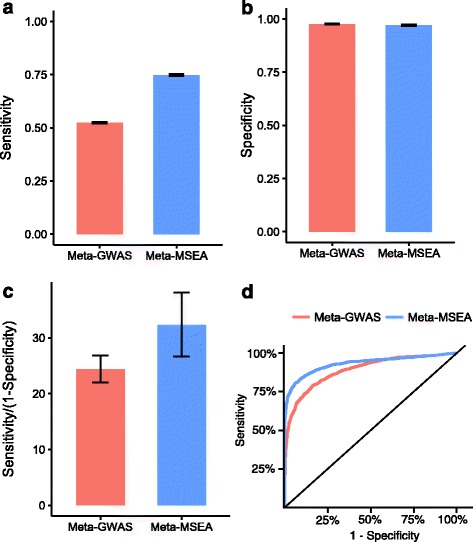
Comparison of performance of SNP-level meta-analysis and pathway-level meta-analysis using simulated gene-sets. Results are produced in the same workflow as stated in Table 1. **a** Sensitivity. **b** Specificity. **c** Positive likelihood ratio (Sensitivity/(1-Specificity)). **d** Receiver operating characteristic curve. Error bars denote the standard error of simulation results

Mergeomics was specifically designed to produce output that is suitable for gene set-level meta-analysis (detailed in Methods). In particular, the reported *P*-values from permutation analysis are always greater than zero, and can be converted to Z-scores by using the inverse Gaussian density function. To demonstrate the practical benefits of Meta-MSEA, we applied Meta-MSEA to the three cholesterol GWAS used in the calibration analysis, and then compared the results with those from the marker-level meta-analysis of the GWAS (denoted as meta-GWAS). While retaining the same-level of specificity, Meta-MSEA showed better sensitivity, positive likelihood ratio and larger area under the ROC curve (Fig. 4). These results suggest that the gene set-level meta-analysis is more powerful than the traditional marker-centric approach to meta-analysis when investigating perturbations to biological processes.

### Weighted key driver analysis (wKDA) to detect disease regulators

#### Rationale and design

The MSEA and meta-MSEA components of Mergeomics identify pathways or co-regulated gene sets that are perturbed in a disease. However, these analyses do not provide information on the detailed interactions between disease genes or help choose which gene to pursue in further mechanistic studies. To provide the answers, the key driver analysis (KDA) was previously developed to detect important hub genes, or key drivers, whose network neighbourhoods are over-represented with disease associated genes [23, 31]. The key driver concept is based on the projection of the disease-associated gene sets onto a network model of gene regulation that represents molecular interactions in the full system (Fig. 2a). However, the original KDA ignored the edge weight information generated by most network inference algorithms. As edge weight typically represents association strength or reliability of the connection between genes, this data carries valuable topological information. Here, we introduce wKDA, a new algorithm that takes into account edge weights to increase accuracy (Fig. 2). Briefly, the edge weights are encoded as local node strengths in the neighbourhood of a hub, and then aggregated to estimate an effective membership score for a disease-associated pathway (technical details in Methods). This approach serves two purposes: firstly, the key driver scores can be quickly recalculated after permuting the node labels thus enabling the empirical estimation of the null distribution and, secondly, the key driver score takes the local connectivity into account when evaluating the impact of a node. wKDA starts by searching a network for candidate hub genes and ignores genes with few connections. It then collects the neighbouring genes for each candidate hub, and estimates the contribution of the disease-associated genes within the neighbourhood of the hub. If the contribution is stronger than what would be expected by chance, we conclude that the hub is a key driver of the disease-associated gene sets (Fig. 2a).

If a subnetwork of genes has multiple highly interconnected genes at the center, it is critical to consider them collectively due to the inherent topological redundancy. For practical purposes, we developed the co-hub concept for wKDA (Fig. 2b) by selecting one of the central genes as the independent hub, and the rest as co-hubs. The rationale is two-fold: first, the statistical power is increased by only considering the independent hubs when adjusting *P*-values, as they also capture the signals from their respective co-hubs. Second, the co-hub concept is a useful qualitative measure when selecting the most promising subnetworks and key drivers for experimental validation. For instance, if a key driver has co-hubs with known functions, these can give clues as to the role of poorly understood genes. On the other hand, if a key driver is to be perturbed in an experiment, it may be important to incorporate the co-hubs as integral parts of the experimental design.

#### Performance evaluation of wKDA and comparison with KDA

To evaluate the performance of wKDA in comparison to the unweighted KDA, we used the reproducibility of KDs of a given gene set mapped to independently constructed gene networks as the performance measure. We first set up three disease-associated gene sets as the test gene sets. These included two lipid subnetworks (denoted as Lipid I and Lipid II) derived from a previous study [23] and the R-HSA-556833 (Metabolism of lipids and lipoproteins) pathway from Reactome. To identify KDs of these test gene sets, we also set up four gene regulatory networks of two tissues (2 independent networks per tissue). The gene-gene interaction network models were probabilistic Bayesian gene regulatory networks constructed from multiple adipose and liver datasets (Additional file 1: Table S3). We organized these networks into two independent weighted adipose networks and two independent weighted liver networks using non-overlapping datasets, where edge weight represents the estimated reliability of an edge between genes.

**Fig. 5:**
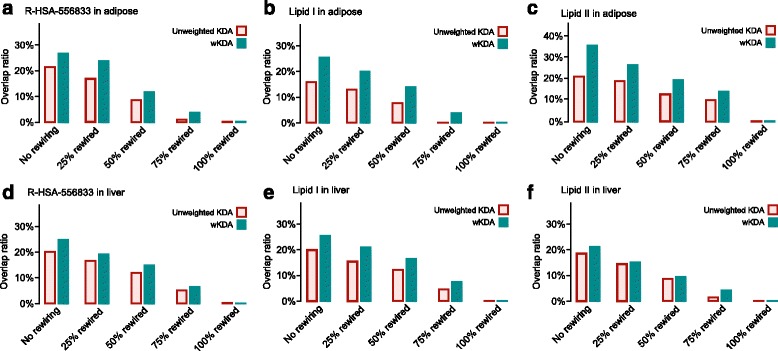
Performance comparison between wKDA and the unweighted key driver analysis. Two empirical subnetworks (Lipid I & II) were obtained from a previous publication [23], and a canonical metabolism of lipids and lipoproteins pathway was obtained from the Reactome database (R-HSA-556833). The methods were tested by projecting the three functional subnetworks onto two independent adipose networks (**a**–**c**) and two independent liver regulatory networks (**d**–**f**). The adipose and liver networks were constructed from a collection of Bayesian tissue-specific network models (Additional file 1: Table S3). Overlap between the tissue-specific key driver signals across two independent regulatory networks was defined according to the Jaccard index. Overlap ratio was calculated for both original networks and networks with 25, 50, 75 or 100 % rewiring of edges

We used the Jaccard overlap index of the identified KD genes between the two independent networks of the same tissue to assess the prediction accuracy of wKDA and KDA (detailed in Methods). The higher the proportion of KDs replicated between independent networks using a method, the higher the Jaccard overlap index and the higher the reliability and performance of the method. As shown in Fig. 5, the new wKDA outperformed the unweighted KDA for all three test gene sets against independent networks in both tissues. To test the sensitivity of the key driver approach, we also partially randomized the adipose and liver networks as a model of topological noise. As expected, when some of the edges were randomly rewired, the number of consistent key drivers between two independent networks of the same tissue declined, and when all edges were rewired, no consistent key drivers were detected (Fig. 5). Notably, wKDA was able to detect consistent signals even when half the network was rewired, thus demonstrating the inherent robustness of the wKDA concept compared to the unweighted version. Importantly, because wKDA was specifically designed for weighted networks whereas the unweighted KDA mainly focuses on the network topology without considering weight information, key drivers with high-weight (i.e., high reliability) edges between subnetwork genes were preferred by wKDA. This difference likely explains the better reproducibility of wKDA compared to the unweighted KDA.

### Case study 1: Application of Mergeomics to multiple cholesterol datasets from different cohorts

In the first case study, we applied the entire Mergeomics pipeline (MSEA, Meta-MSEA, wKDA) to integrate multiple association studies of the same data type with functional genomics and gene networks. We utilized the three cholesterol GWAS from the Finnish, Framingham and GLGC studies described in previous sections, and performed MSEA on individual studies followed by Meta-MSEA across studies. Table 1 lists the top pathways from Meta-MSEA, and the full results are available in Additional file 3. Meta-MSEA yielded more significant *P*-values than those obtained from the pathway analysis of conventional SNP-level meta-GWAS, which was consistent with the simulated signals in the calibration tests. Importantly, when we only included the Finnish and Framingham studies, the two smaller GWAS in Meta-MSEA, the signals for the top pathways were comparable to GLGC, which has 6 times larger samples size than Finnish and Framingham combined.

**Table 1:** Top 15 pathways associated with cholesterol levels out of 1346 canonical pathways tested in three GWAS datasets

Pathway	MSEA			Meta-MSEA		Meta-GWAS
	Finnish (*n* = 8330)	Framingham (*n* = 7572)	GLGC (*n* = 100184)	Without GLGC	With GLGC	
Lipid digestion, mobilization and transport	4.16	5.46	6.15	8.67	13.76	5.00
Lipoprotein metabolism	4.67	4.82	5.94	8.59	13.49	5.41
Chylomicron-mediated lipid transport	4.88	4.87	4.72	8.85	12.61	5.03
Metabolism of lipids and lipoproteins	3.15	1.71	6.15	4.00	8.53	3.56
Cytosolic tRNA aminoacylation	3.58	2.09	1.92	4.77	5.86	2.70
Binding and Uptake of Ligands by Scavenger Receptors	1.88	2.29	3.36	3.46	5.86	2.92
Scavenging by Class A Receptors	1.83	2.22	3.22	3.33	5.62	3.47
Metabolism	1.83	1.48	3.94	2.65	5.36	2.98
PPARA Activates Gene Expression	1.66	2.22	2.83	3.17	5.13	1.33
Retinoid metabolism and transport	1.01	2.75	3.04	2.84	4.94	1.42
Regulation of Lipid Metabolism by Peroxisome proliferator-activated receptor alpha (PPARalpha)	1.32	2.02	2.79	2.64	4.52	1.60
Fatty acid, triacylglycerol and ketone body metabolism	1.48	1.65	2.49	2.49	4.13	1.56
Clathrin derived vesicle budding	1.91	1.27	2.36	2.50	4.05	1.30
Diseases associated with visual transduction	1.41	1.89	2.18	2.62	4.03	2.34
ABC transporters	1.77	0.89	3.16	1.97	4.01	2.75

The results are listed as − log_10_ *P*-values, and the full table is available in Additional file 3. MSEA was run with top 50 % of markers and LD cutoff r^2^ < 50 %. The column ‘Meta-GWAS’ was estimated according to inverse-variance meta-analysis of the cohort specific *P*-values at individual SNP level, followed by MSEA. The Bonferroni-adjusted 5 % significance level for 1346 independent tests is at − log_10_ *P* = 4.43

We observed 82 significant pathways with a Meta-MSEA *P*-value < 0.05. The top hits included major lipoprotein and lipid transport pathways and the receptors that mediate lipid transfer to and from lipoprotein particles. Interestingly, we also found ‘Cytosolic tRNA aminoacylation’ and ‘PPAR-alpha activates gene expression’, suggesting that these transcriptional regulatory processes are intrinsically intertwined with the traditional concepts of enzyme-driven metabolic pathways in cholesterol biosynthesis and transport. Because of the presence of overlaps in gene memberships between certain curated pathways, we merged the 82 significant pathways into 43 non-overlapping gene “subnetworks” at a Jaccard index cutoff of 20 %, and performed a second run of Meta-MSEA using these merged subnetworks to retrieve the top six subnetworks (Additional file 1: Table S4). The strongest signal was observed for Subnetwork 1 (*P* < 10^−16^) that contained genes encoding key apolipoproteins and lipid transport proteins (such as *LDLR*, *CETP* and *PLTP*). Subnetwork 2 (*P* < 10^−8^) included genes related to lipid biosynthesis and catabolism (including the statin target *HMGCR*), oxidoreductive enzymes, metalloproteins and mitochondria. Subnetwork 3 represents a biologically intriguing connection between circulating cholesterol and the immune system: it contained proteins that are involved in the transport of fatty acids and lipids in blood (Albumin and apolipoproteins A1, B, A and L1), collagen genes and the immunoglobulin family. Subnetwork 4 mainly contained the ATP-binding cassette family of transmembrane transporters responsible for lipid and cholesterol transfer across cell membranes. Subnetwork 5 included genes for metabolizing retinoid, an important mediator of cholesterol transport and Subnetwork 6 may reflect the connection between transcriptional regulation and fatty acid metabolism.

**Fig. 6:**
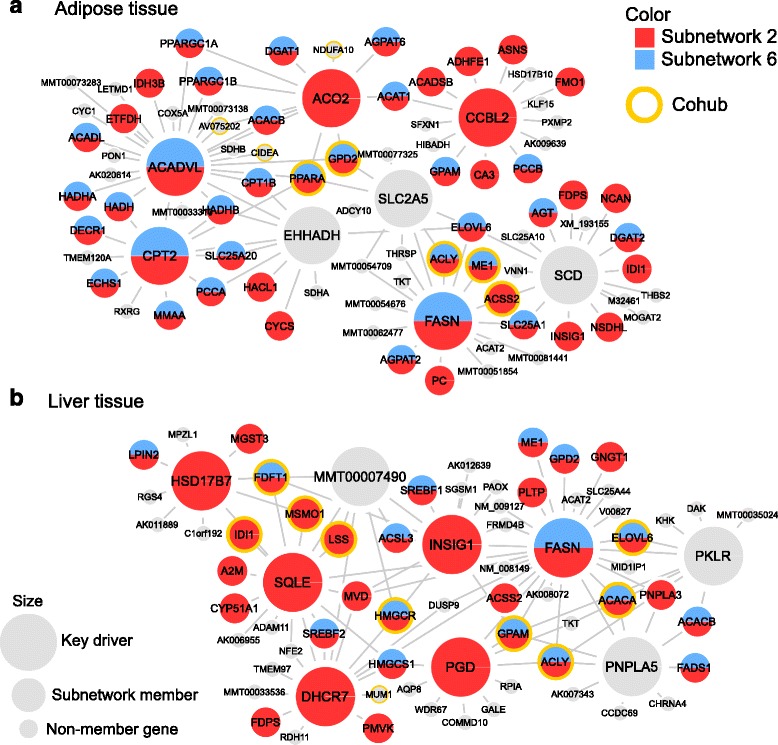
Visualization of adipose (**a**) and liver (**b**) networks around top key drivers that were identified for cholesterol-associated subnetworks. Top key drivers (nodes with the largest size) are selected as the top five independent key regulatory genes (genes whose neighbourhood has less than 25 % overlap with the neighbourhood of other independent hubs) for subnetwork 2 and subnetwork 6. Subnetwork member genes are denoted as medium size nodes and non-member genes as small size nodes. Top co-hubs (co-hubs with FDR < 10^−10^ in wKDA) are highlighted by yellow circles. Only edges that were supported by at least two studies are drawn

Next, we investigated if specific genes could be the key drivers for the aforementioned processes. We applied wKDA to overlay the six cholesterol-associated subnetworks onto gene regulatory networks in liver and adipose tissue. The top five key drivers and their co-hubs are listed in Table 2 with examples of visualization in Fig. 6, and the full results are available in Additional file 4. The top adipose key driver for Subnetwork 2 was *ACADVL* (very long chain acyl-CoA dehydrogenase), which catalyzes the first step in mitochondrial beta-oxidation (Fig. 6a). Notably, the two co-hubs for *ACADVL* (*PPARA* and *CIDEA*) are also highly relevant genes for maintaining lipid homeostasis: *PPARA* is one of the master regulators of lipid metabolism with clinically approved class of drugs (fibrates) already in use; *CIDEA* has been linked to apoptosis, and mouse knock-outs have demonstrated significant effects on the metabolic rate and lipolysis [32]. In the liver (Fig. 6b), the top key driver of Subnetwork 2 was *FASN* (fatty acid synthase), which was a key driver in adipose tissue as well. The second top key driver *SQLE* (squalene epoxidase) and its co-hubs *FDFT1, IDI1, MSMO1, NSDHL, HMGCS1* and *ALDOC* either catalyze or regulate cholesterol biosynthesis. *HMGCR,* although not listed as top five key drivers, was a highly significant key driver (*P* < 10^−14^). Subnetwork 2 and Subnetwork 6 shared multiple common key drivers in the adipose network (Fig. 6a). These included *ACO2* (aconitase 2), an enzyme that catalyzes citrate to isocitrate in mitochondria, and *ACADVL* and its co-hubs. Perturbations to most of the top key drivers, including *ACADVL, FASN, SCD, ACO2, COL1A2, POSTN, EHHADH, DHCR7, HSD17B7, GC, AQP8, INSIG1,* cause abnormal cholesterol and lipid homeostasis according to the Mouse Gene Informatics database and the International Mouse Phenotyping Consortium [33, 34]. In summary, both literature and experiments support the fundamental role of the key drivers in regulating cholesterol metabolism.

**Table 2:** Key drivers for cholesterol-associated gene subnetworks

Subnetworks	−log_10_ P	Functional annotation	Top adipose KDs			Top liver KDs		
			Key driver	−log_10_ P	Co-hubs	Key driver	−log_10_ P	Co-hubs
Subnetwork 1 Lipoprotein	16.0	Lipid transport; cholesterol metabolism; lipoprotein; blood plasma	-	-	-	*SPRY4*	9.5	*ABCG8*
						*S100A10*	4.5	*-*
Subnetwork 2 Lipid metabolism	8.1	Lipid metabolism; metalloprotein; oxidoreductase; endoplasmic reticulum	*ACADVL*	33.7	*PPARA, CIDEA*	*FASN*	49.0	*GPAM, ACLY*
			*FASN*	26.8	*ME1, ACSS2, ACLY, ELOVL6*	*SQLE*	37.4	*FDFT1, IDI1, MSMO1, NSDHL, HMGCS1, ALDOC*
			*SCD*	24.0	*DNMT3L*	*DHCR7*	26.9	*PMVK, MUM1, FDPS, LSS, RDH11, MVD*
			*CCBL2*	23.3	*-*	*HSD17B7*	23.9	*-*
			*ACO2*	23.0	*AV075202, GPD2, NDUFV1*	*MMT00007490*	18.8	*HMGCR, LSS, FDFT1, MVD, ACSL3*
Subnetwork 3 Immunoglobulin	6.1	Immunoglobulin V-set	*COL1A1*	12.4	-	*COL6A3*	21.4	*-*
			*COL1A2*	9.4	*COL3A1,COL2A1, MFAP2*	*VIM*	11.0	*-*
			*OLFML3*	8.8	*-*	*CCDC3*	10.4	*OLFML3*
			*POSTN*	8.3	*COL2A1*	*CXCR7*	9.9	*-*
			*FN1*	7.2	*-*	*FBLN2*	9.0	*-*
Subnetwork 4 ABC transport	5.0	ATP-binding cassette genes	-	-	-	*SPRY4*	12.0	*ABCG8*
						*MMT00062095*	4.3	*-*
						*S100A10*	3.2	*-*
Subnetwork 5 Retinoid metabolism	4.5	Retinoid metabolism; Visual transduction	-	-	-	*GC*	11.2	*RBP4,APOH*
						*TFPI2*	3.2	*-*
						*AQP8*	2.9	*-*
Subnetwork 6 Transcription	3.8	Transcription regulation; fatty acid metabolism; acyltransferase	*SLC2A5*	18.2	*-*	*PKLR*	23.6	*MMT00060232, ELOVL6*
			*ACADVL*	17.7	*PPARA, CIDEA*	*PNPLA5*	19.0	*ACLY, ACACA, PNPLA3*
			*CPT2*	15.9	-	*PGD*	12.2	*-*
			*EHHADH*	15.1	-	*FASN*	11.6	*GPAM,ACLY*
			*ACO2*	13.7	*AV075202, GPD2, NDUFV1*	*INSIG1*	10.7	*-*

Initially, canonical pathways were evaluated for the enrichment of genetic perturbations to circulating cholesterol. As these pathways overlap with each other, non-redundant “subnetworks” were constructed that represent the most shared core genes between overlapping pathways. To verify the association with cholester, enrichment was re-evaluated for the subnetworks (second column in the table). Statistical significance was estimated as described in Table 1. Functional annotations were determined with the DAVID Bioinformatics Tool [45]. Key drivers and co-hubs were determined with the wKDA module within Mergeomics. Bayesian networks from multiple mouse studies were combined to create weighted adipose and liver consensus networks [43, 44]. Gene symbols were translated to human when available

### Case study 2: Application of Mergeomics to glucose datasets of various data types and species

The second case study demonstrates the integrated use of human and mouse resources with diverse data types, and it provides insights into the genes involved in glucose metabolism. The human data came from a GWAS of fasting glucose that included 46,186 non-diabetic participants [35]. The mouse data came from the Hybrid Mouse Diversity Panel (HMDP), and comprised a GWAS [36], TWAS [36] and EWAS [37] of glucose. The HMDP datasets were derived from genotyping, gene expression profiling, epigenome profiling and clinical phenotyping of 100 mouse strains.

The Meta-MSEA approach was applied to all the human and mouse association studies. The top hits captured important glucose homeostasis pathways including glycolysis/gluconeogenesis, beta-cell regulation, incretin homeostasis, adipocytokine signalling and glucose transport (Table 3, full results in Additional file 5). The results also implicated mechanistic connections between lipid metabolism and glucose level based on the findings of carbohydrate-responsive element-binding protein (ChREBP), steroid biosynthesis and lipid transport. Moreover, we highlighted alpha-linolenic acids, an essential fatty acid, whose role in glucose control and metabolic health is under active investigation [38]. When comparing the pathway signals across datasets, it is noticeable that the mouse studies yield relatively weaker association strength. This could be partly explained by the tissue-specificity of HMDP data, as gene expression, methylation and eQTLs used in our analysis were all from the liver tissue, which could have missed pathways in non-hepatic tissues. Despite the weak power of the mouse datasets, 8 out the 13 top pathways demonstrated stronger significance across studies than in the human GWAS alone (Table 3). These results (and the earlier example of circulating cholesterol) demonstrate how Mergeomics was able to identify important biological signals that are subtle in any isolated omics dataset, but consistent across multiple data types and species.

**Table 3:** Pathways associated with fasting glucose across human and mouse association datasets

Pathway	MSEA				Meta-MSEA	
	Human	Mouse	Mouse	Mouse		
	GWAS	GWAS	TWAS	EWAS	Value	FDR
Glycolysis/Gluconeogenesis	2.56	0.88	3.84	0.63	4.73	2.22 %
Starch and sucrose metabolism	3.67	1.37	3.29	0.17	4.57	2.22 %
Regulation And Function Of ChREBP in Liver	3.10	0.93	2.74	0.41	4.08	3.60 %
Nuclear Receptors in Lipid Metabolism and Toxicity	5.58	0.48	1.99	0.35	4.00	3.60 %
Regulation of gene expression in beta cells	4.16	1.75	1.48	0.19	3.97	3.60 %
Type II diabetes mellitus	2.11	1.09	1.48	1.08	3.66	6.00 %
Integration of energy metabolism	2.42	0.33	2.17	1.09	3.34	10.82 %
Steroid biosynthesis	1.10	2.04	1.27	0.76	3.10	14.34 %
alpha-linolenic acid (ALA) metabolism	3.40	0.32	1.72	0.69	3.09	14.34 %
Incretin Synthesis, Secretion and Inactivation	3.24	1.14	0.09	2.06	3.02	14.34 %
Adipocytokine signaling pathway	2.55	0.41	1.13	1.26	2.94	14.34 %
Chylomicron-mediated lipid transport	0.43	0.89	2.89	1.26	2.92	14.34 %
Glucose transport	5.57	1.31	0.27	0.29	2.92	14.34 %

The results are listed as − log_10_ *P*-values, and the full table is available in Additional file 5. MSEA was run with top 50 % of markers, and an LD cutoff r^2^ < 50 % was applied to the GWAS. For the human GWAS, SNPs were assigned to genes based on a 20 kb window in the genome sequence. For the mouse GWAS, liver eQTL data were used for gene assignment. The Bonferroni-adjusted 5 % significance level for 1346 independent tests is at − log_10_P = 4.43

## Conclusions

The explosion of genomics data has shifted the technical challenge from data acquisition to data analysis and interpretation. To respond to the challenge, we developed Mergeomics, a generic pipeline that helps to leverage combined statistical patterns of univariate associations of diverse data types and molecular networks to identify important pathways and key drivers in biological systems. We demonstrated how to use Mergeomics in multi-omics projects with human and animal datasets, and also tested the technical robustness with simulated examples. Through the case studies of cholesterol and glucose regulation, we found that gene networks orchestrated by existing drug targets (such as *PPARA* and *HMGCR*) and less known genes (such as *ACADVL* and collagen genes) potentially regulate circulating cholesterol level, and that both known and novel biological processes likely participate in the genetic and epigenetic regulation of glucose levels. This evidence supports the biological relevance of Mergeomics output. With simulated and real data we demonstrated the robustness of the algorithms in a wide variety of settings and how Mergeomics outperformed other popular tools. Importantly, the inputs to Mergeomics are fully customizable, and accommodate any source dataset that can be represented by i) univariate associations, ii) hierarchical relationships between markers, genes or gene-sets or iii) weighted (gene) networks. Therefore, Mergeomics can guide hypothesis generation across a wide variety of applications.

We acknowledge the following limitations of Mergenomics. First, the current pipeline only takes disease association strength and static information but not directionality and temporal information into consideration, which fortunately covers the majority of available genomics data, but may limit the detection of additional biological signals. Second, although genetic information, when available, can help infer causal relationships, the bioinformatics analyses from Mergeomics mainly serve to generate testable biological hypotheses rather than directly implicating causality. Therefore, the causal roles of the key driver genes, pathways and networks inferred by Mergeomics require explicit experimental validation. Despite the limitations, Mergeomics provides the scientific community with the first open source implementation of a methodology that has a proven track record of successful biomedical applications. Future development of Mergeomics will focus on addressing the limitations and improve its flexibility and performance by incorporating directional information, dynamic time-course data and prediction of potential therapeutic agents.

## Methods

### Market set enrichment analysis

The default setting of MSEA takes as input 1) summary statistics from global marker association studies (e.g., GWAS, EWAS, TWAS), 2) measurement of relatedness or dependency between genomic markers, 3) mapping between markers and genes, and 4) functionally defined gene sets (e.g., biological pathways or co-regulated genes). For GWAS, SNPs are first filtered based on the LD structure to select for only SNPs that are relatively independent given an LD threshold [23] (Details in Additional file 6). For other types of association studies, correlations between co-localized markers may be used. For a given gene set, gene members are first mapped to markers based on the mapping file and then the disease association p values of the corresponding markers are extracted to test for enrichment of association signals based on the following null hypothesis:

*Given the set of all distinct markers from a set of N genes, these markers contain an equal proportion of positive association study findings when compared to all the distinct markers from a set of N random genes*

We only focus on distinct markers to reduce the effect of shared markers among gene families that are both close in the genome and belong to the same pathway (and presumably have overlapping functionality). Furthermore, our software has a feature that merges genes with shared markers before analysis to further reduce artefacts from shared markers. MSEA uniquely adopts a hierarchical gene-based permutation which estimates the expected distribution of the test statistic under the null hypothesis by randomly shuffling the gene labels while retaining the assignment of mapped markers to genes, preserving the hierarchical marker-gene-pathway cascade (Additional file 2: Figure S4). As an alternative option, the marker labels can also be shuffled to form the null distribution. Both options are offered in the R package.

To avoid assessing enrichment based on any pre-defined association study *p*-value threshold (e.g., *p* < 0.05) which can mean different association strengths in studies of varying sample size and power, we developed a test statistic with multiple quantile thresholds to automatically adapt to any dataset:

$$ \chi ={\displaystyle \sum_{i=1}^n}\frac{O_i-{E}_i}{\sqrt{E_i}+\kappa } $$

In the formula, *n* denotes the number of quantile points, *O* and *E* denote the observed and expected counts of positive findings (i.e. signals above the quantile point), and κ = 1 is a stability parameter to reduce artefacts from low expected counts for small gene sets. The frequency of permuted signals that exceed the observation is determined as the enrichment *P*-value. For highly significant signals where the frequency-based value is zero (i.e. no permuted signal exceeds the observation), we fit a parametric model to the simulated null distribution to approximate the corresponding Z-score (details in Additional file 6). For meta-MSEA of multiple association studies, pathway enrichment Z-scores from each dataset are first estimated with MSEA. The meta P value is then estimated by integrating individual Z-scores using the Stouffer’s method [39].

### Weighted key driver analysis

wKDA utilizes both the network topology information and the edge weight information of a molecular network when available (illustrated in Additional file 2: Figure S5). In wKDA, a network is first screened for suitable hub genes whose degree (number of genes connected to the hub) is in the top 25 % of all network nodes (Additional file 2: Figure S5, middle box on the left). We further classify these genes as either independent hubs or co-hubs, where a co-hub is defined as a gene that shares a large proportion of its neighbours with an independent hub (Details in Additional file 2: Figure S5). Once the hubs and co-hubs have been defined, the disease-associated gene sets that were discovered by MSEA or meta-MSEA are overlaid onto the molecular network to see if a particular part of the network is enriched for the disease genes. First, the edges that connect a hub to its neighbours are simplified into node strengths (strength = sum of adjacent edge weights) within the neighbourhood (Plots B-D in Additional file 2: Figure S5), except for the hub itself. For example, the top-most node in Plot C has three edges that connect it with the other neighbours with weights that add up to 7 in Plot D. By definition, the hub at the center will have a high strength which will skew the results, so we use the average strength over the neighbourhood for the hub itself. The reduction of the hub neighbourhood into locally defined node strengths improves the speed of the algorithm and makes it easier to define an enrichment statistic that takes into account the local interconnectivity. In particular, the weighting of the statistic with the node strengths emphasizes signals that involve locally important genes over isolated peripheral nodes. In Plot D of Additional file 2: Figure S5, the overlap between the hub neighbourhood and a hypothetical disease-associated gene set is indicated by the circles around the top three nodes. The sum of the strengths of the disease genes is 15, which represents 57 % of the total sum of 26.4 in the neighbourhood (pie chart in Plot D). The final enrichment score is estimated as described below.

The null hypothesis for the enrichment of disease genes within a subnetwork can be expressed as

*Weighted key driver H*_*0*_ *: Given the set of nodes adjacent to a key driver, and with each node having a local strength as estimated by the mutual connectivity, the ratio of the sum of strengths of disease genes to the total sum of strengths of all genes in the key driver subnetwork is equal to the ratio for a randomly selected gene set that matches the number of disease genes.*

The test statistic for the wKDA is analogous to the one used for MSEA

$$ \chi =\frac{O-E}{\sqrt{E}-\kappa } $$

except that the values *O* and *E* represent the observed and expected ratios of disease genes in a hub neighbourhood. In particular,

$$ E=\frac{N_k{N}_p}{N} $$

is estimated based on the hub degree *N*_*k*_, disease gene set size *N*_*p*_ and the order of the full network *N*, with the implicit assumption that the weight distribution is isotropic across the network.

Statistical significance of the disease-enriched hubs, henceforth key drivers, is estimated by permuting the gene labels in the network and estimating the *P*-value based on the simulated null distribution. To control for multiple testing, we perform adjustments in two tiers. First, the *P*-values for a single subnetwork are multiplied by the number of independent hubs (Bonferroni adjustment). All hubs with adjusted *P* > 1 are discarded. For random data, the truncated results will be uniformly distributed between 0 and 1, and hence they can be treated as regular *P*-values. In the second stage, all the *P*-values for the subnetworks are pooled and the final FDRs are estimated by the Benjamini-Hochberg method [40].

### MSEA performance evaluation

MSEA can be reconfigured depending on the type of dataset and study design. We identified several parameters that could affect the performance of the pipeline such as marker filtering by including top disease/trait associated markers based on a percentage cutoff, marker dependency or relatedness (such as LD) cutoff for pruning redundant markers, and the mapping between genes and markers. Here we focus on the marker filtering percentage and marker dependency cutoff as they represent the two key technical challenges. Of note, the mapping between genes and markers can be defined empirically [10, 12], but we used a chromosomal distance-based approach for testing to make Mergeomics consistent with most of the existing pathway enrichment tools. In fact, for GWAS, the assignment of SNPs to their target genes based on their chromosomal location is the commonly adopted approach in other methods, whereas Mergeomics allows users to apply any available assignment method, including the data from tissue-specific eQTL studies and ENCODE.

To evaluate the performance of MSEA in independent datasets, we collected GWAS summary data for circulating cholesterol from 7572 individuals in the Framingham Study [25], 8330 Finnish individuals [24], and 100,184 participants from the Global Lipid Genetics Consortium [26]. Cholesterol metabolism and transport is one of the most studied and understood areas of human biology, which makes cholesterol GWAS [26] an informative dataset for method assessment. The Framingham and Finnish studies are completely independent. The GLGC dataset is the largest meta study for cholesterol traits and contains the two smaller studies, but the total overlap was less than 10 % between the datasets. All participants were predominantly Caucasian descent, and we used the corresponding LD data from HapMap [41] and 1000 genomes project [42] in our analyses to remove redundant SNPs in LD. To determine a suitable combination of parameters and to compare performance of different methods, we simulated true positives and true negatives. True positive signals related to cholesterol and lipid metabolism were collected from the Reactome pathway R-HSA-556833, “the metabolism of lipids and lipoproteins”. These genes were grouped into 300 positive control pathways, including 100 with size 25, 100 with size 100, 100 with size 250, respectively. Simultaneously, 300 negative control pathways with the same size distribution as the positive control pathways were generated by randomly selecting genes from the non-cholesterol gene pool consists of 8633 genes from the pathway database of Reactome [29], BioCarta (http://cgap.nci.nih.gov/Pathways/BioCarta_Pathways) and KEGG [30]. These manually generated control pathways were combined with 1346 original canonical pathways for benchmarking.

The control and canonical pathways were analysed by MSEA and two widely-used existing tools MAGENTA and i-GSEA4GWAS. The latter two tools estimate the genetic associations for each gene, and then test if the aggregate gene score for a pathway is higher than expected. MAGENTA identifies the peak disease-associated SNP for each gene, and then adjusts the statistical significance of the peak SNP according to the size of the gene, LD and other potential confounders to produce the gene score. i-GSEA4GWAS uses a similar approach where a gene is considered significant if it contains any of the top 5 % SNPs, and the pathway score is estimated by comparing the observed ratio of significant genes within the pathway against the expected ratio in the full set of genes that were covered by the GWAS. The performance was evaluated as sensitivity (number of positive control pathways at FDR < 25 % divided by total number of positive control pathways), specificity (number of negative control pathways at FDR < 25 % divided by total number of negative control pathways) and the likelihood to pick up true positive pathways (Positive Likelihood Ratio), calculated as sensitivity/(1 − specificity).

Integrated analysis of diverse data types across species was tested in the second case study. The datasets included a human GWAS for fasting glucose on 46,186 non diabetic subjects [35], and mouse GWAS, EWAS and TWAS for fasting glucose from the HMDP, which consists of 100 different mouse strains [36, 37]. The epigenome and transcriptome data were generated from the liver tissues from the mouse strains on standard chow diet, and mouse liver eQTLs were used in gene-SNP assignment for the HMDP GWAS data for consistency. For EWAS data, DNA methylation sites were mapped to adjacent genes based on a chromosomal distance of 50 kb. All other MSEA parameters were the same as those applied in the cholesterol analysis (see the descriptions of the case studies for more information).

### wKDA performance evaluation

We assessed the performance of wKDA based on the robustness of the key driver signals in independent gene networks. We collected Bayesian networks that were constructed from published genomic studies where both DNA and RNA were extracted from adipose and liver tissue samples [43, 44]. The collection of Bayesian networks was split into two independent sets of weighted adipose networks and weighted liver networks from non-overlapping datasets (Additional file 1: Table S3). Edge weights were quantified based on the consistency of the edge between datasets. Using these networks and three test gene sets related to lipid metabolism as inputs, we ran wKDA to identify liver and adipose key drivers of the lipid gene sets. To benchmark the wKDA performance, we compared wKDA with the previously developed unweighted KDA [23, 31]. The prediction accuracy of wKDA and KDA was evaluated by the Jaccard overlap index between the top key driver genes from the two independent networks of each tissue, which represents the proportion of KDs that can be replicated between independent networks. Jaccard overlap index measures the overlap between two KD sets X and Y each containing lists of KD genes, and is calculated based on the following formula: $\mathrm{overlap}\left(\mathrm{X},\kern0.28em \mathrm{Y}\right)=\frac{\left|\mathrm{X}\cap \mathrm{Y}\right|}{\left|\mathrm{X}\cup \mathrm{Y}\right|}$. The higher the overlap or replication rates of KDs detected between two independent network using a KDA method, the higher the Jaccard overlap index and the higher the performance of the corresponding method.

### Availability

Mergeomics is available as a freely downloadable Bioconductor package released under GPL license, version 2 (https://www.bioconductor.org/packages/devel/bioc/html/Mergeomics.html). The package supports full Mergeomics functionality.

## Additional files

Additional file 1: Tables S1–S4. (DOCX 40 kb)
Additional file 2: Figures S1–S5. (PDF 380 kb)
Additional file 3: Full results from pathway-level meta-analysis for MSEA using total cholesterol GWAS from GLGC, Finnish and Framingham. (XLSX 147 kb)
Additional file 4: Results from weighted key driver analysis for six cholesterol associated subnetworks of adipose and liver regulatory networks. (XLSX 402 kb)
Additional file 5: Full results from pathway-level meta-analysis for fasting glucose datasets from human GWAS, mouse GWAS, mouse TWAS and mouse EWAS. (XLSX 136 kb)
Additional file 6: Supplementary notes. (DOCX 14 kb)

## Abbreviations

eQTL: Expression quantitative trait loci
EWAS: Epigenome-wide association study
FDR: False discovery rate
GWAS: Genome-wide association study
HMDP: Hybrid mouse diversity panel
KDA: Key driver analysis
LD: Linkage disequilibrium
MSEA: Marker set enrichment analysis
TWAS: Transcriptome-wide association study
wKDA: Weighted key driver analysis
