# Supplementary material for: Mergeomics: multidimensional data integration to identify pathogenic perturbations to biological systems
Source: BMC Genomics. 2016 Nov 4;17:874. doi: 10.1186/s12864-016-3198-9 (PMC5097440; doi:10.1186/s12864-016-3198-9)
Supplement: Additional file 1: Tables S1–S4. — (DOCX 40 kb) [file 12864_2016_3198_MOESM1_ESM.docx]

**Supplementary Tables**

**Table S1.** Comparison of MSEA performance across multiple combinations of parameters using simulated gene sets.

| **GWAS** | **Marker_inch** | **LD_cutoff** | **Distance** | **Sensitivity**^a^ | **Specificity**^b^ | **Positive Likelihood Ratio**^c^ |
| --- | --- | --- | --- | --- | --- | --- |
| GLGC | 10% | 0.1 | 10kb | 0.705 | 0.976 | 28.959 |
| GLGC | 10% | 0.3 | 10kb | 0.708 | 0.975 | 28.333 |
| GLGC | 10% | 0.5 | 10kb | 0.720 | 0.978 | 32.712 |
| GLGC | 50% | 0.1 | 10kb | 0.637 | 0.980 | 31.867 |
| GLGC | 50% | 0.3 | 10kb | 0.692 | 0.977 | 30.087 |
| GLGC | 50% | 0.5 | 10kb | 0.694 | 0.980 | 35.305 |
| GLGC | 100% | 0.1 | 10kb | 0.570 | 0.983 | 34.200 |
| GLGC | 100% | 0.3 | 10kb | 0.661 | 0.982 | 36.722 |
| GLGC | 100% | 0.5 | 10kb | 0.678 | 0.982 | 37.000 |
| GLGC | 10% | 0.1 | 20kb | 0.721 | 0.977 | 31.348 |
| GLGC | 10% | 0.3 | 20kb | 0.724 | 0.978 | 32.418 |
| GLGC | 10% | 0.5 | 20kb | 0.729 | 0.980 | 37.085 |
| GLGC | 50% | 0.1 | 20kb | 0.661 | 0.983 | 38.154 |
| GLGC | 50% | 0.3 | 20kb | 0.713 | 0.980 | 35.650 |
| GLGC | 50% | 0.5 | 20kb | 0.714 | 0.983 | 42.840 |
| GLGC | 100% | 0.1 | 20kb | 0.581 | 0.986 | 40.535 |
| GLGC | 100% | 0.3 | 20kb | 0.676 | 0.984 | 43.170 |
| GLGC | 100% | 0.5 | 20kb | 0.703 | 0.984 | 43.061 |
| GLGC | 10% | 0.1 | 50kb | 0.686 | 0.980 | 33.738 |
| GLGC | 10% | 0.3 | 50kb | 0.674 | 0.978 | 30.164 |
| GLGC | 10% | 0.5 | 50kb | 0.745 | 0.974 | 28.641 |
| GLGC | 50% | 0.1 | 50kb | 0.595 | 0.981 | 31.298 |
| GLGC | 50% | 0.3 | 50kb | 0.679 | 0.977 | 29.522 |
| GLGC | 50% | 0.5 | 50kb | 0.714 | 0.976 | 29.342 |
| GLGC | 100% | 0.1 | 50kb | 0.563 | 0.984 | 35.167 |
| GLGC | 100% | 0.3 | 50kb | 0.680 | 0.977 | 30.000 |
| GLGC | 100% | 0.5 | 50kb | 0.741 | 0.969 | 23.892 |
| Framingham | 10% | 0.1 | 10kb | 0.013 | 0.999 | 13.000 |
| Framingham | 10% | 0.3 | 10kb | 0.029 | 0.997 | 8.600 |
| Framingham | 10% | 0.5 | 10kb | 0.038 | 0.993 | 5.700 |
| Framingham | 50% | 0.1 | 10kb | 0.006 | 0.999 | 6.333 |
| Framingham | 50% | 0.3 | 10kb | 0.287 | 0.983 | 16.558 |
| Framingham | 50% | 0.5 | 10kb | 0.260 | 0.985 | 16.935 |
| Framingham | 100% | 0.1 | 10kb | 0.001 | 0.999 | 2.000 |
| Framingham | 100% | 0.3 | 10kb | 0.020 | 0.994 | 3.588 |
| Framingham | 100% | 0.5 | 10kb | 0.018 | 0.996 | 4.583 |
| Framingham | 10% | 0.1 | 20kb | 0.001 | 0.999 | 1.333 |
| Framingham | 10% | 0.3 | 20kb | 0.012 | 0.998 | 5.000 |
| Framingham | 10% | 0.5 | 20kb | 0.074 | 0.994 | 11.684 |
| Framingham | 50% | 0.1 | 20kb | 0.002 | 0.999 | 2.500 |
| Framingham | 50% | 0.3 | 20kb | 0.019 | 0.999 | 14.000 |
| Framingham | 50% | 0.5 | 20kb | 0.146 | 0.992 | 19.087 |
| Framingham | 100% | 0.1 | 20kb | 0.001 | 0.999 | 1.500 |
| Framingham | 100% | 0.3 | 20kb | 0.017 | 0.996 | 4.636 |
| Framingham | 100% | 0.5 | 20kb | 0.043 | 0.996 | 10.000 |
| Framingham | 10% | 0.1 | 50kb | 0.007 | 0.999 | 5.000 |
| Framingham | 10% | 0.3 | 50kb | 0.007 | 0.998 | 3.143 |
| Framingham | 10% | 0.5 | 50kb | 0.018 | 0.998 | 9.167 |
| Framingham | 50% | 0.1 | 50kb | 0.009 | 0.995 | 1.733 |
| Framingham | 50% | 0.3 | 50kb | 0.079 | 0.996 | 18.231 |
| Framingham | 50% | 0.5 | 50kb | 0.100 | 0.996 | 23.077 |
| Framingham | 100% | 0.1 | 50kb | 0.010 | 0.998 | 4.429 |
| Framingham | 100% | 0.3 | 50kb | 0.015 | 0.997 | 4.400 |
| Framingham | 100% | 0.5 | 50kb | 0.048 | 0.996 | 12.083 |
| Finnish | 10% | 0.1 | 10kb | 0.000 | 0.999 | 0.500 |
| Finnish | 10% | 0.3 | 10kb | 0.000 | 0.999 | 0.000 |
| Finnish | 10% | 0.5 | 10kb | 0.000 | 1.000 | 1.000 |
| Finnish | 50% | 0.1 | 10kb | 0.001 | 0.999 | 1.500 |
| Finnish | 50% | 0.3 | 10kb | 0.002 | 0.999 | 3.500 |
| Finnish | 50% | 0.5 | 10kb | 0.028 | 0.997 | 9.222 |
| Finnish | 100% | 0.1 | 10kb | 0.000 | 1.000 | N/A |
| Finnish | 100% | 0.3 | 10kb | 0.001 | 1.000 | N/A |
| Finnish | 100% | 0.5 | 10kb | 0.001 | 1.000 | N/A |
| Finnish | 10% | 0.1 | 20kb | 0.078 | 0.992 | 9.320 |
| Finnish | 10% | 0.3 | 20kb | 0.167 | 0.989 | 15.656 |
| Finnish | 10% | 0.5 | 20kb | 0.251 | 0.986 | 17.535 |
| Finnish | 50% | 0.1 | 20kb | 0.275 | 0.984 | 17.574 |
| Finnish | 50% | 0.3 | 20kb | 0.302 | 0.987 | 23.205 |
| Finnish | 50% | 0.5 | 20kb | 0.328 | 0.984 | 20.500 |
| Finnish | 100% | 0.1 | 20kb | 0.022 | 0.999 | 21.667 |
| Finnish | 100% | 0.3 | 20kb | 0.076 | 0.997 | 22.700 |
| Finnish | 100% | 0.5 | 20kb | 0.122 | 0.993 | 16.682 |
| Finnish | 10% | 0.1 | 50kb | 0.215 | 0.987 | 16.100 |
| Finnish | 10% | 0.3 | 50kb | 0.306 | 0.980 | 15.283 |
| Finnish | 10% | 0.5 | 50kb | 0.348 | 0.979 | 16.328 |
| Finnish | 50% | 0.1 | 50kb | 0.573 | 0.962 | 15.212 |
| Finnish | 50% | 0.3 | 50kb | 0.576 | 0.961 | 14.636 |
| Finnish | 50% | 0.5 | 50kb | 0.595 | 0.961 | 15.248 |
| Finnish | 100% | 0.1 | 50kb | 0.497 | 0.967 | 15.204 |
| Finnish | 100% | 0.3 | 50kb | 0.522 | 0.962 | 13.858 |
| Finnish | 100% | 0.5 | 50kb | 0.548 | 0.957 | 12.638 |

^a^ Number of postive control pathways at FDR < 25% / Total number of positive control pathways

^b^ Number of negative control pathways at FDR ≥ 25% / Total number of negative control pathways

^c^ Sensitivity / (1-Specificty)

**Table S2.** Performance comparison of MSEA, MAGENTA and i-GSEA4GWAS across three GWAS datasets using real gene sets.

| **Method** | **Marker**  **Incl**^a^ | **LD cutoff**^b^ | **Finnish**^c^ | **Framingham^d^** | **GLGC**^e^ | **Obs**^f^ | **Exp**^g^ | **True Pos**^h^ | **False Pos^i^** | **Alpha**^j^ |
| --- | --- | --- | --- | --- | --- | --- | --- | --- | --- | --- |
| MSEA | 50% | 50% | 84 | 61 | 77 | 13.00 | 0.22 | 12.78 | 0.13 | 0.046 |
| MAGENTA | n/a | n/a | 114 | 81 | 144 | 6.00 | 0.73 | 5.27 | 0.70 | 0.080 |
| i-GSEA4GWAS | n/a | n/a | 426 | 503 | 32 | 17.00 | 3.80 | 13.20 | 16.31 | 0.230 |

^a^ Proportion of top markers included

^b^ Linkage disequilibrium r^2^ cutoff

^c^ Number of pathways with P < 0.05 from Finnish cohorts

^d^ Number of pathways with P < 0.05 from the Framingham Study

^e^ Number of pathways with P < 0.05 from the Global Lipid Genetics Consortium

^f^ Observed overlap between the top pathways from the three datasets

^g^ Expected overlap for size-matched randomly picked pathways for the three datasets

^h^ Estimated number of true positive signals

^i^ Estimated number of false positives based on the alpha level (see next)

^j^ Estimated actual significance threshold based on the average number of signals across each dataset, and the number of consistent signals

**Table S3.** Data resources of Bayesian networks used in the study.

| **Tissue** | **Independent Bayesian networks set I** | |  | **Independent Bayesian networks set II** | |
| --- | --- | --- | --- | --- | --- |
|  | **Species** | **Dataset** |  | **Species** | **Dataset** |
| **Adipose** | Mouse | Bx129JaxShort* |  | Mouse | BxAJaxShort [[1](#_ENREF_1)] |
|  | Mouse | BxAMCI [[1](#_ENREF_1)] |  | Mouse | BxCast [[2](#_ENREF_2)] |
|  | Mouse | BxDJaxShort |  | Mouse | BxHandHxB [[2](#_ENREF_2)] |
|  | Mouse | BxHapoE [[3](#_ENREF_3), [4](#_ENREF_4)] |  | Mouse | BxHwt [[2](#_ENREF_2)] |
|  |  |  |  | Mouse | CastxB [[2](#_ENREF_2)] |
| **Liver** | Mouse | Bx129JaxShort* |  | Mouse | BxAJaxShort [[1](#_ENREF_1)] |
|  | Mouse | BxAMCI [[1](#_ENREF_1)] |  | Mouse | BxCast [[2](#_ENREF_2)] |
|  | Mouse | BxDJaxlong |  | Mouse | BxHandHxB [[2](#_ENREF_2)] |
|  | Mouse | BxDJaxShort |  | Mouse | BxHwt [[2](#_ENREF_2)] |
|  | Mouse | BxHapoE [[3](#_ENREF_3), [4](#_ENREF_4)] |  | Mouse | CastxB [[2](#_ENREF_2)] |
|  |  |  |  | Mouse | Attie [[5](#_ENREF_5)] |

* Accessed from Sage Synapse Biocuration (https://[www.synapse.org/#!Synapse:syn47391](http://www.synapse.org/#!Synapse:syn47391))

**Table S4.** Results from the 2nd run of pathway-level meta-analysis on 43 non-overlapping supersets merged from 82 significant pathways related to total cholesterol.

| Subnetwork | Annotation^a^ | Module^b^ | P^c^ | Value^d^ |
| --- | --- | --- | --- | --- |
| Subnetworks1 | **Lipoprotein** | **rctm0647,rctm0648,rctm0239** | **9.15E-17** | **16.04** |
| Subnetworks2 | **Lipid metabolism** | **rctm0680,rctm0686** | **8.26E-09** | **8.08** |
| Subnetworks3 | **Immunoglobulin** | **rctm0168,rctm1091** | **8.99E-07** | **6.05** |
| Subnetworks4 | **ABC transport** | **M11911,rctm0007** | **1.13E-05** | **4.95** |
| Subnetworks5 | **Retinoid metabolism** | **rctm0332,rctm1360,rctm1050** | **2.98E-05** | **4.53** |
|  |  | **M13088** | **1.49E-04** | **3.83** |
| Subnetworks6 | **Transcription** | **rctm0415,rctm0844,rctm1003** | **1.50E-04** | **3.82** |
|  |  | **rctm0876** | **3.37E-04** | **3.47** |
|  |  | **rctm0223** | **9.01E-04** | **3.05** |
|  |  | M311 | 1.14E-03 | 2.94 |
|  |  | rctm0678,rctm0250,rctm1418,rctm0516,rctm0654 | 2.16E-03 | 2.67 |
|  |  | rctm0331 | 2.91E-03 | 2.54 |
|  |  | rctm0987,rctm0059 | 4.95E-03 | 2.31 |
|  |  | M17377 | 5.44E-03 | 2.26 |
|  |  | rctm0171,M9488 | 5.87E-03 | 2.23 |
|  |  | rctm1081 | 6.08E-03 | 2.22 |
|  |  | M17294 | 6.68E-03 | 2.18 |
|  |  | M16121,M1749,M5436 | 6.76E-03 | 2.17 |
|  |  | rctm1417,M14691,rctm0293 | 1.25E-02 | 1.90 |
|  |  | M4741 | 1.54E-02 | 1.81 |
|  |  | rctm0941,rctm0111,rctm0940 | 1.79E-02 | 1.75 |
|  |  | M1519 | 1.82E-02 | 1.74 |
|  |  | rctm0929 | 2.00E-02 | 1.70 |
|  |  | M3812 | 2.25E-02 | 1.65 |
|  |  | M2529,M1909,M9043,M11520,M5489,M2623,M7860 | 2.33E-02 | 1.63 |
|  |  | rctm0709 | 2.43E-02 | 1.61 |
|  |  | M18615 | 2.47E-02 | 1.61 |
|  |  | M7399 | 2.64E-02 | 1.58 |
|  |  | rctm0177 | 2.68E-02 | 1.57 |
|  |  | rctm0102,rctm0762 | 2.72E-02 | 1.57 |
|  |  | rctm0508,rctm1200 | 3.08E-02 | 1.51 |
|  |  | M2404 | 3.16E-02 | 1.50 |
|  |  | M15997,M15394,M6487 | 3.33E-02 | 1.48 |
|  |  | rctm1014 | 3.66E-02 | 1.44 |
|  |  | M2288,M19784,M1908,M9494,M9664,M7739,M3430 | 4.06E-02 | 1.39 |
|  |  | rctm0233 | 4.14E-02 | 1.38 |
|  |  | M11266 | 4.47E-02 | 1.35 |
|  |  | M8601 | 4.89E-02 | 1.31 |
|  |  | rctm0489,rctm0488 | 5.26E-02 | 1.28 |
|  |  | M11106 | 5.91E-02 | 1.23 |
|  |  | M4629 | 6.37E-02 | 1.20 |
|  |  | M2044 | 6.82E-02 | 1.17 |
|  |  | M648 | 7.24E-02 | 1.14 |

Modules and merged subnetworks that have Bonferroni corrected P < 0.05 are shown in bold.

^a^ Functional annotation determined using DAVID[[6](#_ENREF_6)]

^b^ ID of modules merged into the subnetwork

^c^ Meta-MSEA p-values

^d^ -log10 transformed meta-MSEA p-values

**Supplementary References**

1. Derry JM, Zhong H, Molony C, MacNeil D, Guhathakurta D, Zhang B, Mudgett J, Small K, El Fertak L, Guimond A *et al*: **Identification of genes and networks driving cardiovascular and metabolic phenotypes in a mouse F2 intercross**. *PloS one* 2010, **5**(12):e14319.

2. Schadt EE, Molony C, Chudin E, Hao K, Yang X, Lum PY, Kasarskis A, Zhang B, Wang S, Suver C *et al*: **Mapping the genetic architecture of gene expression in human liver**. *PLoS biology* 2008, **6**(5):e107.

3. Wang SS, Schadt EE, Wang H, Wang X, Ingram-Drake L, Shi W, Drake TA, Lusis AJ: **Identification of pathways for atherosclerosis in mice: integration of quantitative trait locus analysis and global gene expression data**. *Circulation research* 2007, **101**(3):e11-30.

4. Yang X, Schadt EE, Wang S, Wang H, Arnold AP, Ingram-Drake L, Drake TA, Lusis AJ: **Tissue-specific expression and regulation of sexually dimorphic genes in mice**. *Genome research* 2006, **16**(8):995-1004.

5. Tu Z, Keller MP, Zhang C, Rabaglia ME, Greenawalt DM, Yang X, Wang I-M, Dai H, Bruss MD, Lum PY: **Integrative analysis of a cross-loci regulation network identifies App as a gene regulating insulin secretion from pancreatic islets**. 2012.

6. Huang DW, Sherman BT, Lempicki RA: **Systematic and integrative analysis of large gene lists using DAVID bioinformatics resources**. *Nature protocols* 2009, **4**(1):44-57.
