# Supplementary material for: Mergeomics: multidimensional data integration to identify pathogenic perturbations to biological systems
Source: BMC Genomics. 2016 Nov 4;17:874. doi: 10.1186/s12864-016-3198-9 (PMC5097440; doi:10.1186/s12864-016-3198-9)
Supplement: Additional file 2: Figures S1–S5. — (PDF 380 kb) [file 12864_2016_3198_MOESM2_ESM.pdf]

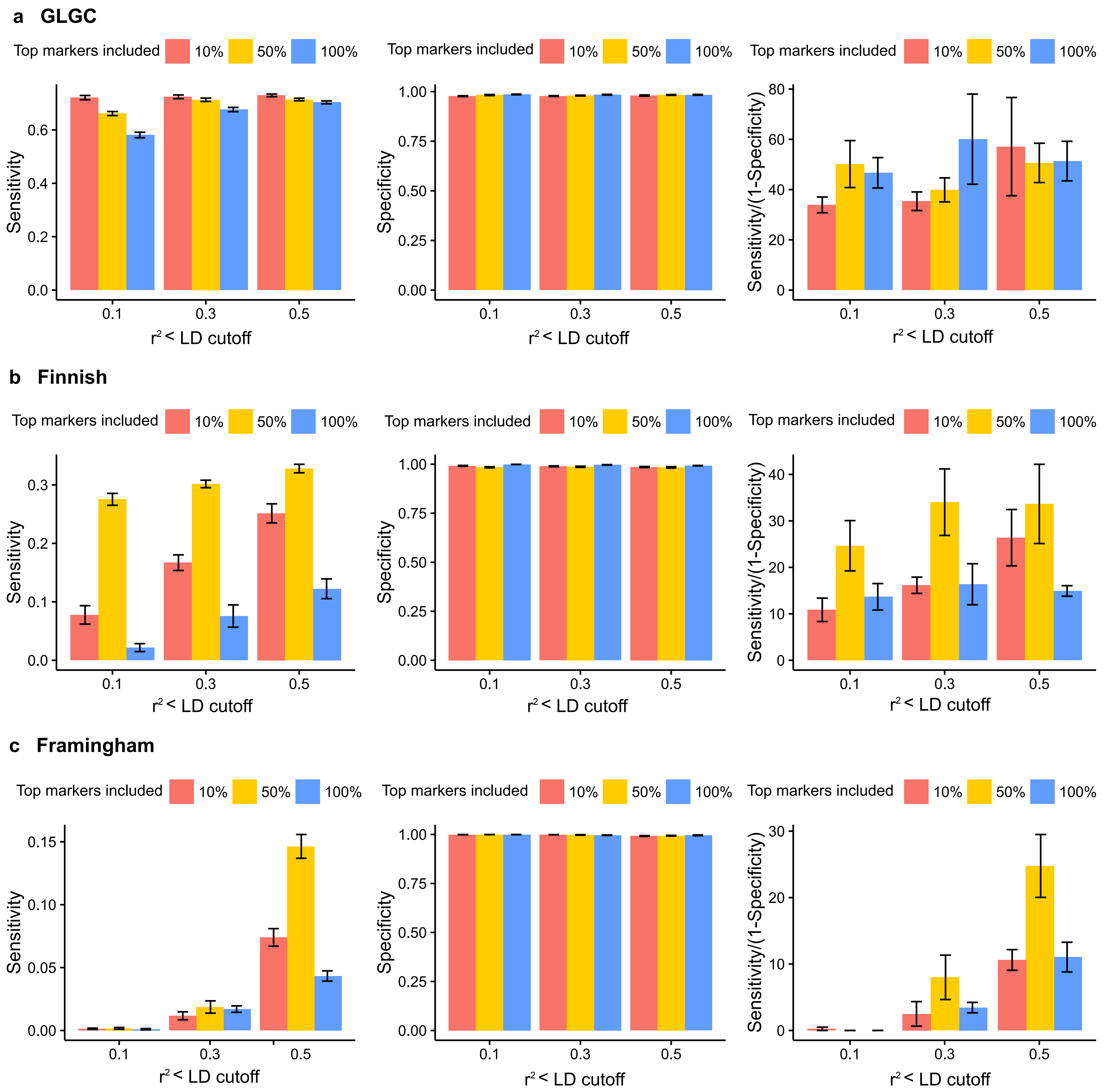

Figure S1: Sensitivity, specificity and positive likelihood ratios of MSEA in capturing simulated lipid genesets across combinations of GWAS datasets, top markers included and LD cutoffs. (**a**) Results from GLGC GWAS. (**b**) Results from Finnish GWAS. (**c**) Results from Framingham GWAS. 20kb window distance mapping is used for all analyses.

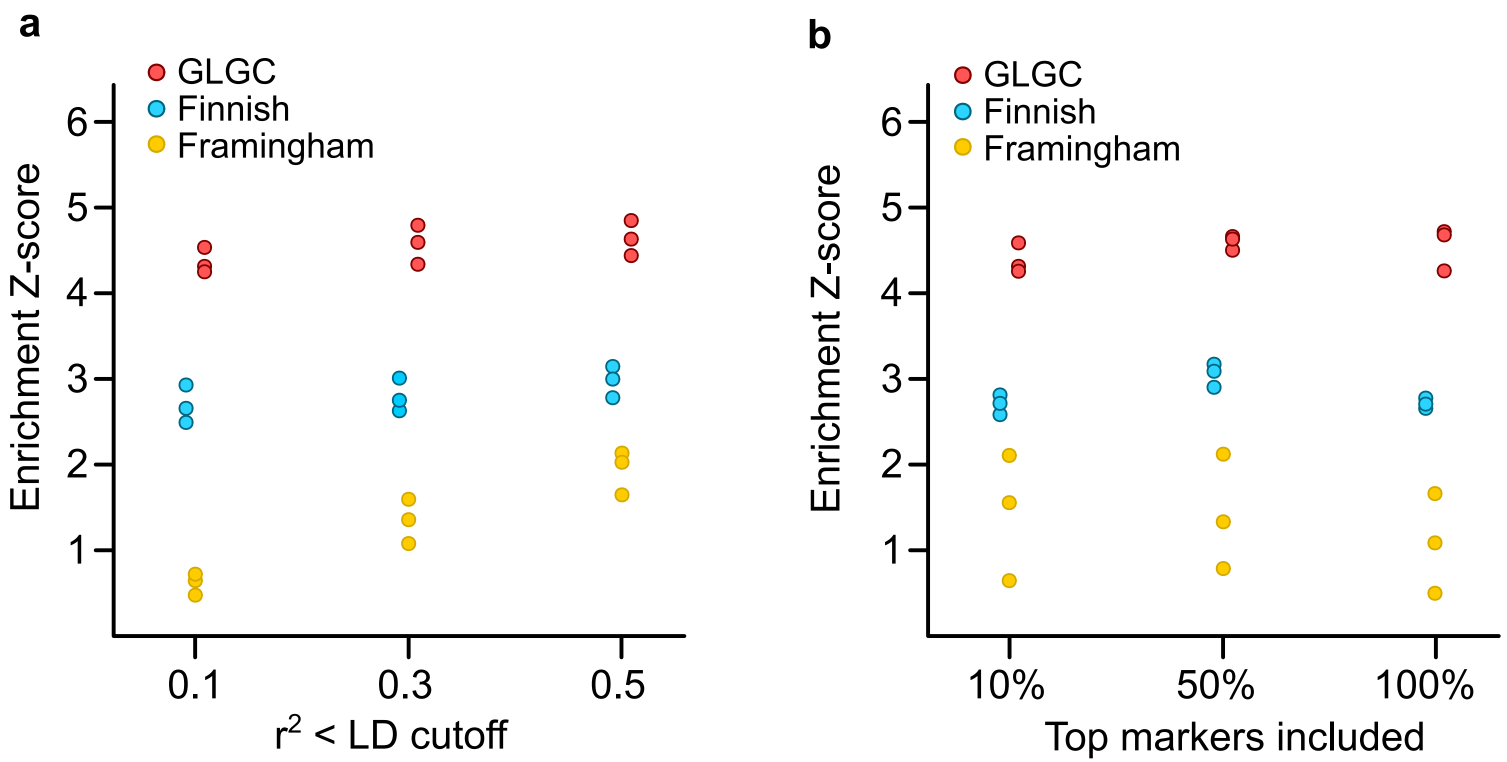

Figure S2: MSEA signals of lipid homeostasis genes for combinations of linkage disequilibrium pruning parameter and marker filtering (top markers included). Results are from 20kb window distance mapping. Z-scores of dots with the same color in the LD cutoff plot (**a**) represent the values from different marker filtering settings, and vice versa in (**b**).

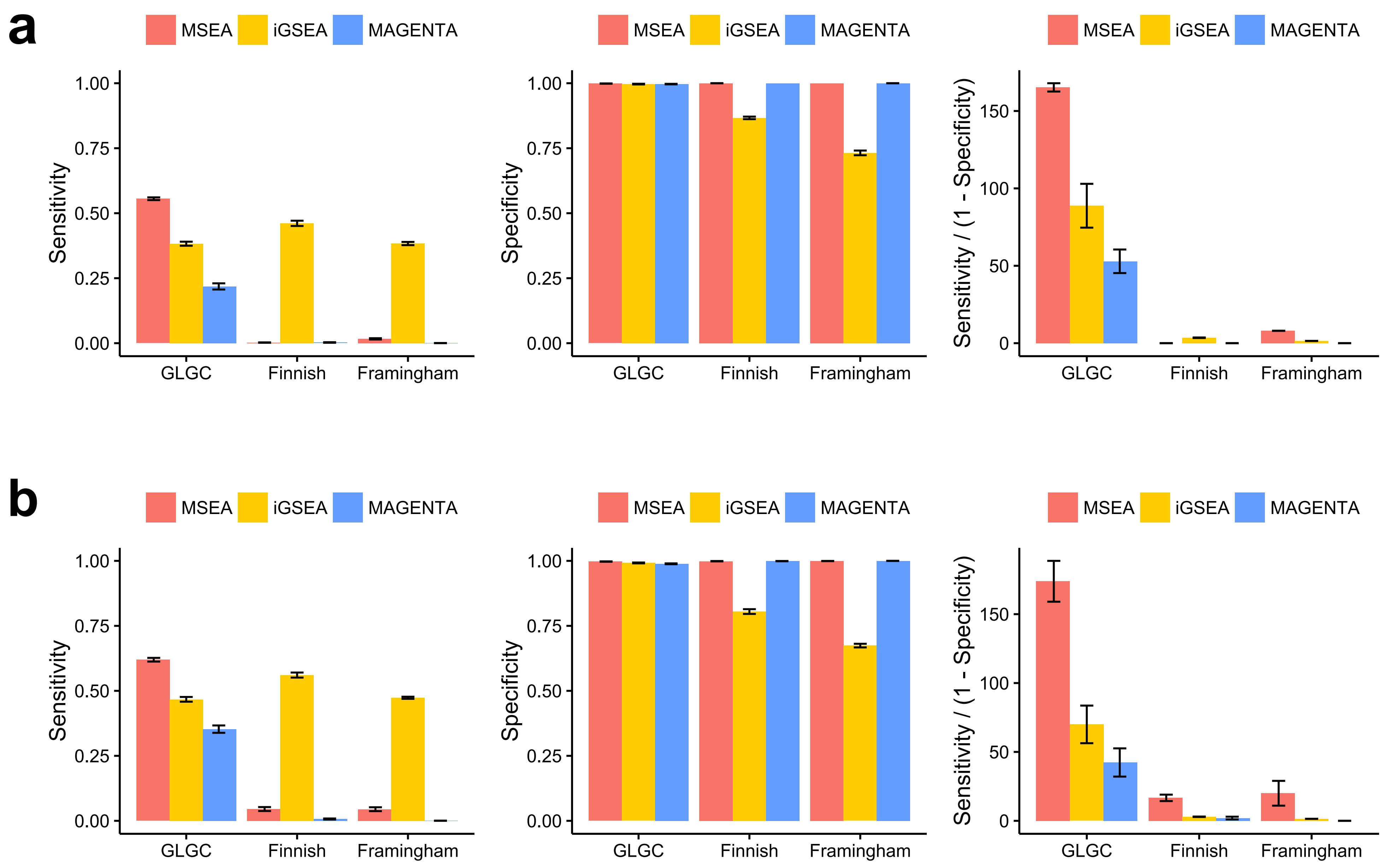

Figure S3. Performance comparison of MSEA, iGSEA and MAGENTA at FDR 5% (a) and FDR 10% (b).

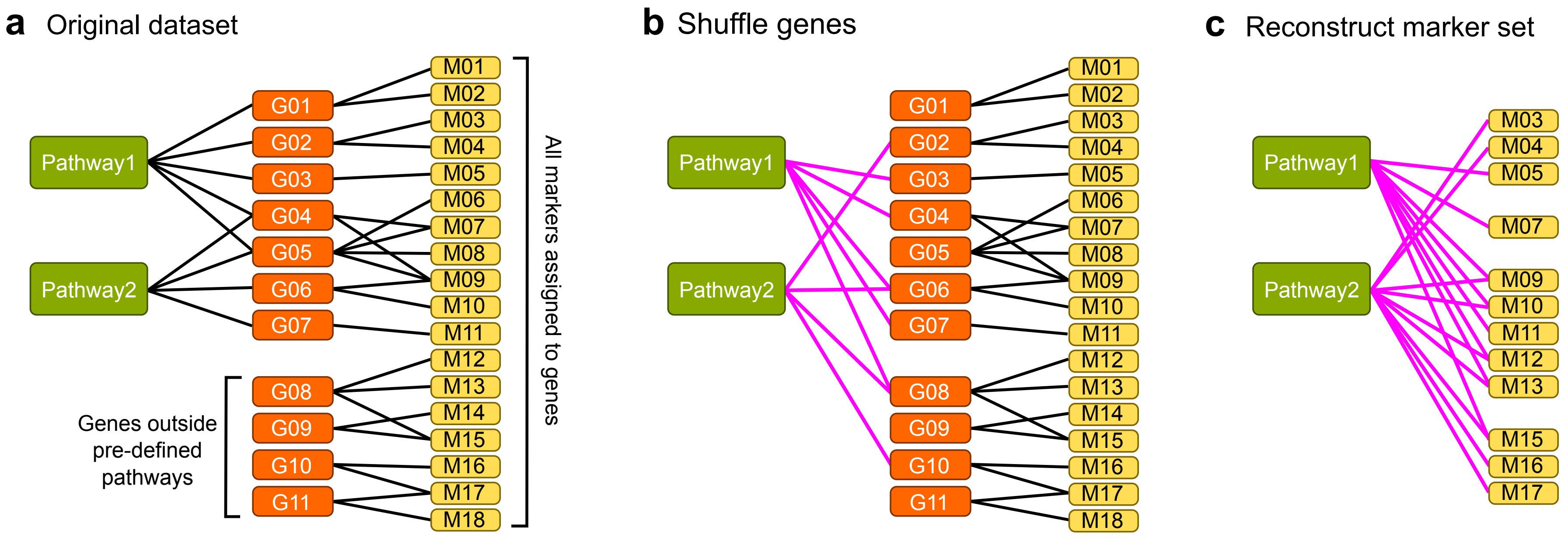

Figure S4: Schematic illustration of the hierarchical structure in genetic datasets (a) and the randomization procedure that was used in the gene-permuted MSEA (b & c).

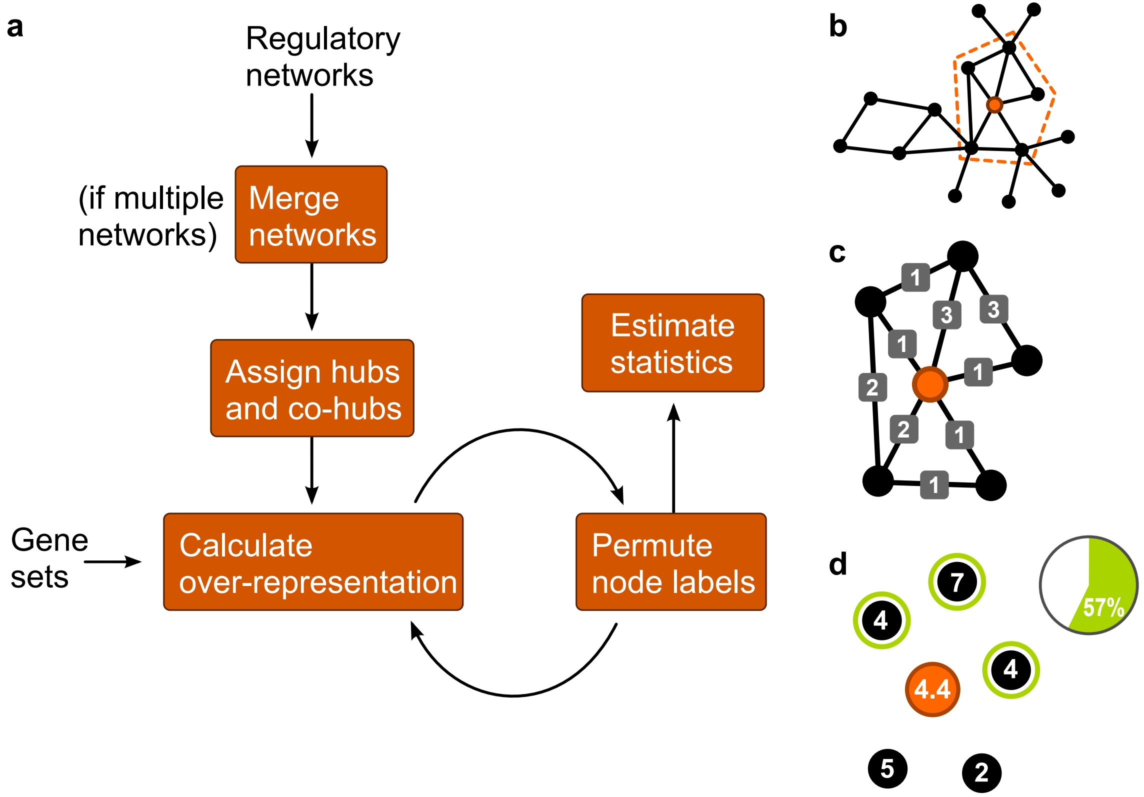

Figure S5: Schematic illustration of the weighted key driver analysis (a) and the key driver enrichment statistic (b-d).
