# Supplementary material for: Mergeomics: multidimensional data integration to identify pathogenic perturbations to biological systems
Source: BMC Genomics. 2016 Nov 4;17:874. doi: 10.1186/s12864-016-3198-9 (PMC5097440; doi:10.1186/s12864-016-3198-9)
Supplement: Additional file 6: — Supplementary notes. (DOCX 14 kb) [file 12864_2016_3198_MOESM6_ESM.docx]

## Supplementary Notes

## Linkage disequilibrium filtering:

The LD filtering algorithm comprises two phases: first, the SNPs are sorted by the disease associations (GWAS data) to prioritize the most important loci. The second phase starts by the selection of the top SNP. The next highest ranked SNP is then compared against the first one to see if LD is acceptable. If not, the SNP is discarded. The same step is then repeated for the remaining list while always checking if the next SNP is in LD with any of the already accepted ones. Subsequently, all accepted SNPs are guaranteed not to be in LD with each other above a designated LD threshold.

## Definition of co-hubs in wKDA

The candidate independent hubs are first sorted according to the node degree, from low to high. This is to ensure that we capture local structures rather than one master hub that covers the majority of the network (e.g. housekeeping genes would make poor drug targets due to global side-effects). Next, the sorted hubs are tested one by one for neighbourhood overlaps with the already accepted hubs. If sufficient overlap (as defined under section “definition of overlap between two gene sets” below, default value is 33%) is detected, the current hub is assigned as a co-hub for the previously accepted overlapping hub.

## Adaptive Gaussian approximation for estimating P-values in MSEA and wKDA

The exact shape of the null distribution is dependent on the size of the gene set and on the mapping between the genes and the markers (MSEA) or on the size and topology of the gene network (wKDA). To estimate the P-value from these various permutation approaches, we created a generic algorithm for a parametric approximation using the Gaussian function. In the range where a direct frequency-based P-value is accurate (i.e. with 10,000 permutations it is possible to accurately estimate P-values above ~0.001), we found that the Gaussian approximation was highly concordant. For P < 0.001, we found that the Gaussian model produced biologically plausible rankings of statistical significance. We tested other models, but found that the potential benefit from using more long-tailed distributions was outweighed by the difficulties in applying them in practice. For instance, the t-distribution was more conservative than the Gaussian estimate, but assigning an appropriate degree of freedom was problematic given the diverse nature of the null hypotheses.

Let X denote the series of simulated test statistics (as defined in the previous section) from the permutation analysis. Then the transformation algorithm can be expressed as

1) α = min(X_0_), X_1_ = X_0_ - α
2) β = median(X_1_), X_2_ = X_1_ / β
3) X_3_ = log(γX_2_ + 1)
4) μ = mean(X_3_), σ = sd(X_3_)
5) Evaluate how well X_3_ approximates N(μ, σ)
6) If necessary, try a different γ and go back to Step 3.

The parameters from Steps 1-4 can be saved and reapplied to new data, which makes it possible to determine the transformation exclusively based on simulated statistics, and then apply it to the observed test statistic to yield the parametric enrichment score

$$Z_{N}=\frac{\log\left( \gamma\left( X-\alpha\right)\beta^{-1}+1 \right)-\mu}{\sigma}$$

The rationale for Gaussian approximation is based on the attractive analytical properties of Gaussian distributions. Nevertheless, if the approximation is inaccurate, the results can be biased and lead to erroneous conclusion. In particular, any dependencies between markers tend to elongate the tails of the “true” distribution when using marker permutations for the MSEA. For this reason, we also report the raw frequency of false positive findings from the permutation analysis for each gene set.
